# HEPACAM1 and 2 are differentially regulated in canine mammary adenomas and carcinomas and its lymph node metastases

**DOI:** 10.1186/1746-6148-6-15

**Published:** 2010-03-15

**Authors:** Robert Klopfleisch, Patricia Klose, Afonso da Costa, Leo Brunnberg, Achim D Gruber

**Affiliations:** 1 Department of Veterinary Pathology, Freie Universität Berlin, Robert-von-Ostertag-Straße 15, 14163 Berlin, Germany; 2 Department of Veterinary Medicine Small Animal Clinic, Freie Universität Berlin, Oertzenweg 19b, 14163 Berlin, Germany

## Abstract

**Background:**

Cell adhesion is an important regulator of cell growth and motility. Recently the hepatocyte cell adhesion molecules 1 and 2 (HEPACAM1 and 2), members of the immunoglobulin family of adhesion genes, have been identified. HEPACAM1 is involved in negative cell cycle regulation via p53, p21 and p27 signalling but also mediates increased human breast cancer cell spread. The role and expression pattern of HEPACAM2 has not been analyzed so far. In the present study we quantified gene expression levels of HEPACAM1 and 2 to evaluate their possible role during the carcinogenesis of canine mammary tumours.

**Results:**

Adenomas displayed increased HEPACAM1 and 2 mRNA expression levels and decreased HEPACAM1 protein expression levels when compared to normal gland, carcinomas and lymph node metastases. In contrast, metastatic carcinomas, intravascular tumour cells and lymph node metastases had HEPACAM 1 protein and mRNA expression levels similar to normal gland but decreased HEPACAM2 mRNA expression when compared to normal gland of the same dog.

**Conclusions:**

HEPACAM1 and 2 seem to be important for cell-cell adhesion of normal and neoplastic canine mammary cells. The loss of HEPACAM1 protein expression in adenomas but not in carcinomas questions its role as a tumour suppressor at late stages of malignant transformation and indicates that it might rather be involved in physiologic mammary cell adhesion and canine mammary tumour metastasis. Furthermore, it can be speculated, whether HEPACAM2 plays a different role in malignancy and metastasis of canine mammary tumours since its transcriptional levels are different in carcinomas and their lymph node metastases when compared to HEPACAM1.

## Background

Cell adhesion to neighbouring cells and the extracellular matrix is a dynamic process and essential for the maintenance of the cellular differentiation status. In contrast, metastatic progression of epithelial tumours is regularly associated with loss of cell-cell contact and a decrease in cell differentiation. The same is obviously true for canine mammary tumours that regularly show metastatic spread to distant organs including the lung. It is therefore not surprising that invasive character and metastatic spread of canine mammary tumours (CMT) is associated with decreased expression of cell adhesion associated genes like E-Cadherin, Connexin 26, 43 and Paxillin [1-3]. However, these genes not only act as a cellular anchor but also strongly influence cellular proliferation and migratory status through complex downstream signalling cascades [4,5].

Recently we found a significant decrease in expression levels of the cell cycle inhibitor p27 in metastatic canine mammary carcinomas and their lymph node metastases [6,7]. So far, decreased expression of transforming growth factor β (TGFβ) family members has been identified as a possible cause of decreased p27 expression levels and increased cellular proliferation of canine mammary tumours [8]. However, due to the ambivalent anti-proliferative and growth stimulatory roles of TGFβ at different stages of mammary tumour carcinogenesis other upstream regulators may be involved in the release of cell cycle brakes like p27 [9]. The hepatocyte cell adhesion molecule 1 (HEPACM1) has been identified as a link between cell cycle arrest at the G2/M junction via p21, p27 and p53 mediated mechanisms in a breast cancer cell line [10]. However, HEPACAM1 also modulates cell-extracellular matrix interaction and supports breast cancer cell spread [11]. On the transcriptional level HEPACAM1 expression is decreased in several human cancer types including breast cancer but protein expression was not analyzed so far [10]. The role of the HEPACAM family member 2 (HEPACAM2) in normal and neoplastic mammary epithelial cells is unknown.

Due to the findings in human tissues we hypothesized that HEPACAM1 expression might be involved in the decreased p27 expression levels in canine mammary tumours. In the present study we therefore characterized the differential mRNA expression of HEPACAM1 and 2 in laser microdissected frozen tissue samples and HEPACAM1 protein expression in normal mammary gland epithelium, adenomas and metastatic canine mammary carcinomas, intravascular tumour cells and lymph node metastases.

## Results

### mRNA and protein expression levels of HEPACAM1 in normal gland, primary tumours, intravascular tumour cells and lymph node metastases

**Figure 1:**
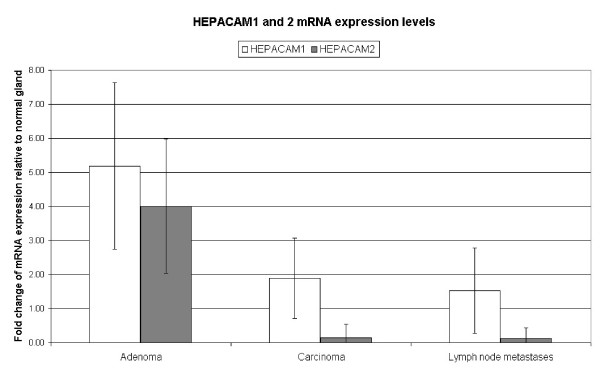
**Comparison of average fold change in HEPACAM1 and HEPACAM2 mRNA expression in canine mammary adenomas, carcinomas and lymph node metastases (IVTC = Intravascular tumour cells)**.

HEPACAM1 mRNA expression was significantly increased in adenomas when compared to normal gland, carcinomas and lymph node metastases (Table 1, Figure 1). Specifically, nine out of ten adenomas had mRNA expression levels higher than 2.0 fold of the normal gland of the same dog. In contrast, HEPACAM1 mRNA expression was not significantly changed in carcinomas and their lymph node metastases when compared to normal gland (Table 1, Figure 1).

**Table 1:** mRNA and protein expression levels of HEPACAM1 and 2 in tissue samples of non-neoplastic canine mammary gland and canine mammary tumours

Gene	Normal gland	Adenoma	Carcinoma	IVTC	Lymph node metastases
Average fold change in mRNA expression levels relative to normal gland of the same dog					
HEPACAM1*	1	5.2 (SD ± 2.4)	1.9 (SD ± 1.2)	-	1.5 (SD ± 1.2)
HEPACAM2*	1	4.0 (SD ± 2.0)	0.1 (SD ± 0.4)	-	0.1 (SD ± 0.3)

Percentage of immunohistochemical HEPACAM positive tissue samples					
HEPACAM1	100%	13%	94%	95%	89%

IVTC: Intravascular tumour cells

* Significant differences (*p *< 0.05) in mRNA expression levels were observed between microdissected adenomas/carcinomas and adenomas/lymph node metastases (ANOVA)

**Figure 2:**
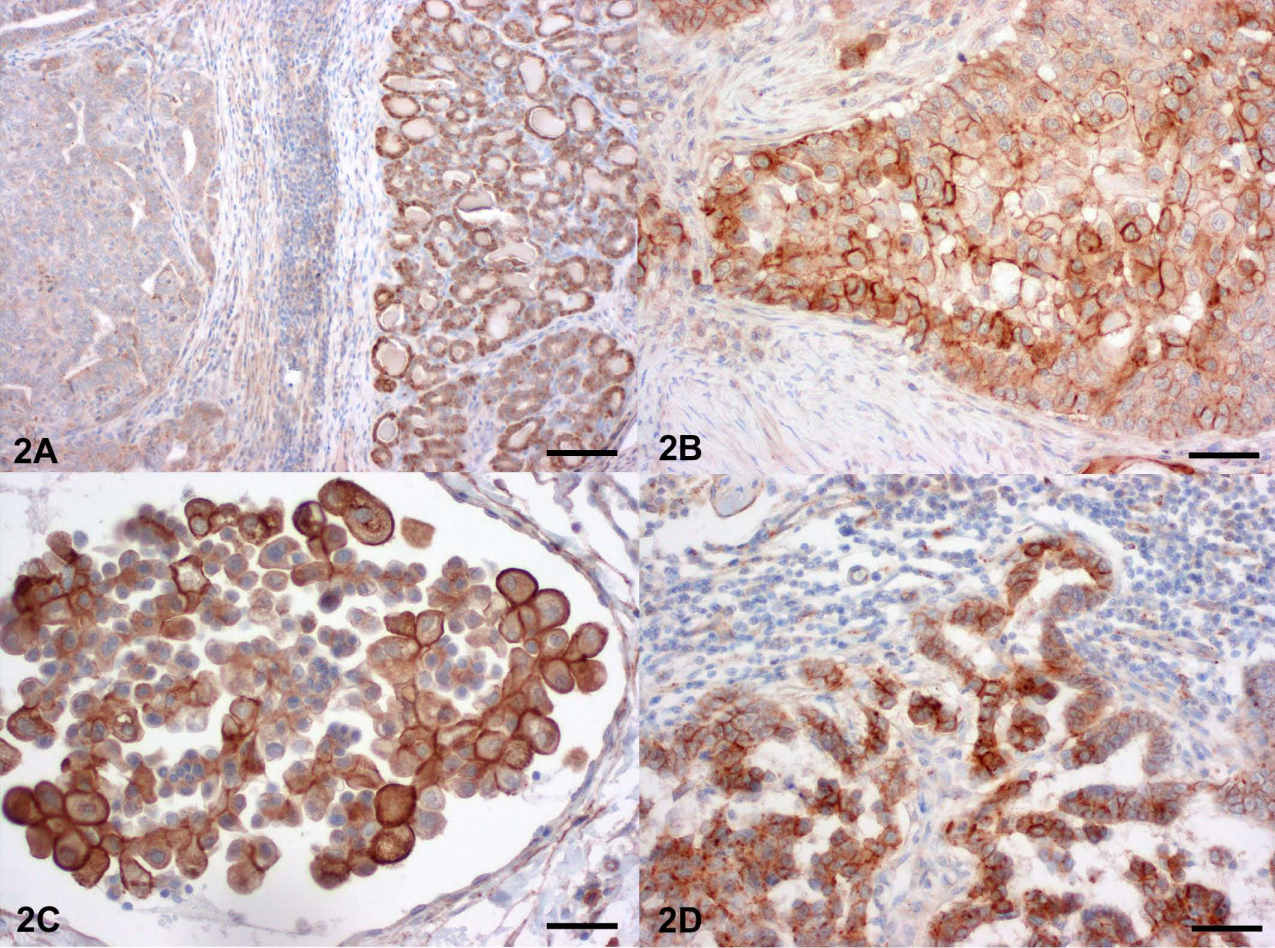
**Mammary gland, dog. Immunohistochemical detection of HEPACAM1 protein**. (A) Non neoplastic mammary gland with membrane-bound expression of HEPACAM1 (right) and adenoma with complete loss of HEPACAM1 expression (left). Bar = 1250 μm. (B) In contrast, almost all carcinomas expressed membrane-bound HEPACAM1. Bar = 250 μm (C) Intravascular tumour cells expressed HEPACAM1 at their cell membrane. Bar = 200 μm (D) The majority of tumour cells lymph node metastases of mammary carcinomas expressed membrane bound HEPACAM1 with increased expression at sites of cell to cell contact. Bar = 300 μm Haematoxylin counterstain.

HEPACAM1 protein was expressed at the cell membranes of all normal gland specimens (Table 1, Figure 2A). Furthermore, 94% of carcinomas, 95% of intravascular tumour cells and 89% lymph node metastases had HEPACAM1 protein expression in more than 10% of cells (Figure 2B, C, D). Surprisingly, only 13 percent of adenomas had membrane-bound HEPACAM1 protein expression (Table 1, Figure 2A), despite their increased HEPACAM1 transcription levels.

### mRNA expression levels of HEPACAM2 in normal gland, primary tumours and metastases

HEPACAM2 mRNA expression was significantly increased in microdissected adenomas when compared to normal gland, carcinomas and lymph node metastases (Table 1, Figure 1). In detail, seven out of ten adenomas had mRNA expression levels higher than 2.0 fold of the normal gland HEPACAM2 expression of the same dog. In contrast, 12 out of 13 carcinomas and 13 out of 13 lymph node metastases had decreased HEPACAM2 mRNA expression levels lower than 0.5 fold of the normal gland epithelium of the same dog (Table 1, Figure 1).

## Discussion

The exact mechanisms of canine mammary tumour metastasis are unknown and this is one reason that there are currently no treatment options available for this tumour type except excision of the primary tumour. In the present study we therefore asked whether HEPACAM1 is a potential tumour suppressor in canine mammary gland and whether its expression is decreased similar to human breast cancer. Furthermore, we aimed at identifying the HEPACAM2 expression levels at different stages of malignant progression of canine mammary gland epithelium.

Normal mammary gland, carcinomas and metastatic cells constantly expressed membrane-bound HEPACAM1. In contrast, almost none of the adenomas expressed HEPACAM1 protein despite increased HEPACAM1 mRNA expression levels. These surprising findings raise several questions regarding potential roles of HEPACAM1 during malignant transformation of canine mammary tumours. Assuming that the HEPACAM1 protein expressed in carcinomas is functionally active, the role of HEPACAM1 as a tumour suppressor via p21, p27 and p53 in canine mammary tumours is questionable. On the contrary, it can be hypothesized that HEPACAM1 expression is associated with increased metastatic spread and cell motility as has been shown for human breast cancer cells before [12]. It will be interesting to analyze in an additional study whether non-metastatic carcinomas differ from metastatic carcinomas by decreased expression levels of HEPACAM1 protein. However, HEPACAM 1 obviously has also a physiologic role in normal mammary gland cell adhesion and maybe in cell cycle control since virtually all normal gland epithelial cells express the protein.

The differences in HEPACAM1 mRNA and protein expression levels in adenomas when compared to carcinomas indicate that decreased HEPACAM1 protein levels in adenomas is caused by failure of mRNA translation or increased proteolysis. However, decreased protein expression levels may induce increased HEPACAM1 transcription in adenomas in a feedback mechanism, although other so far unknown transcription inducers may also cause the increased mRNA expression levels.

The function and expression pattern of the expected immunoglobulin-like, membrane protein HEPACAM2 has not been analyzed in any species so far. In the present study, we found an increased HEPACAM2 expression in adenomas when compared to normal gland. In contrast, carcinomas and lymph node metastases had a constant and marked decrease in HEPACAM2 expression levels. HEPACAM1 and 2 mRNA expression levels are therefore similar in adenomas, while carcinomas and metastases have decreased HEPACAM2 but not HEPACAM1 mRNA expression levels. It can therefore be speculated that HEPACAM2 expression decreases with increasing malignancy of canine mammary tumours as is usually observed for cell-adhesion genes in a wide variety of tumours types. However, evaluation of the protein expression pattern is needed to support the findings for HEPACAM2 on the transcriptional level. Unfortunately, HEPACAM2 specific antibodies are not available so far for any species.

## Conclusions

A significant decrease in HEPACAM1 protein expression was found in canine mammary adenomas. In contrast, HEPACAM1 protein was constantly expressed in normal mammary gland, carcinomas, intravascular tumour cells and metastatic cells. The data question the role of HEPACAM1 as an effective tumour suppressor at late stages of canine mammary carcinogenesis. In contrast, the HEPACAM1 expression pattern makes it more likely that the protein is involved in physiologic cell adhesion of the canine mammary epithelium and also in metastatic spread of canine mammary tumours. HEPACAM2 transcription levels were decreased in mammary carcinomas but further analysis of the function and the expression pattern of the HEPACAM2 protein are needed to evaluate its role in normal mammary gland physiology and malignant transformation.

## Methods

### Dogs and tissue processing

Thirty simple, tubulopapillary and solid carcinomas with metastases in the regional lymph node, thirty simple adenomas and the normal mammary gland of the same dogs but from a different mammary complex were included in the study. For 13 simple, tubulopapillary and solid carcinomas, 13 lymph node metastases of the same dogs, 10 simple adenomas and the 23 normal glands of all dogs, fresh frozen tissue specimens were available from intact female dogs in anestrus and proestrus and used for laser microdissection and mRNA isolation. These tissues were snap frozen in liquid nitrogen at -80°C within 15 min after resection and stored until further use. Paraffin sections (2 μm) of all tissues were routinely stained with hematoxylin and eosin and tumours were classified according to the World Health Organization (WHO) [13]. The study was approved by the local animal welfare officer and informed consent was obtained from patient owners. No additional harm was caused by the study and tissue samples were surgically removed based solely to improve the animal health.

### Laser-capture microdissection and reverse transcription

To obtain highly specific microdissected tumour cell samples without contamination by other cells types, five consecutive sections of 6-8 μm thickness from the frozen tissue samples were mounted on glass slides covered with a polyethylene naphthalate membrane (PALM Microlaser Technologies). Sections were fixed for 2 min in 95% ethanol at -20°C and stained with hematoxylin and eosin in diethylpyrocarbonate (DEPC)-treated water. Sections were then dehydrated in ascending graded ethanol (30, 50, 70, 99%). Approximately, 5 × 10^6 ^μm^2 ^were excised from tissue sections of normal mammary gland, adenomas, carcinomas and lymph node metastases. Cells were laser pressure catapulted into the caps of 0.5-ml reaction tubes containing 20 μL of lysis buffer (NucleoSpin RNA XS; Macherey&Nagel). Total RNA was extracted and purified using a commercial kit (NucleoSpin RNA XS; Macherey&Nagel) including DNAse digestion. Total RNA was reverse transcribed using the iScript cDNA synthesis kit (Biorad).

### Quantitative real-time polymerase chain reaction (qRT-PCR)

Primer sequences for HEPACAM1, HEPACAM2 and the housekeeping genes hypoxanthine-phosphoribosyl transferase (HPRT), ATP-synthase subunit 5B (A5B), ribosomal protein L32 (RPL32) are shown in Table 2. Real-time qRT-PCR and data analyses were performed using the MX 3000P Quantitative PCR System and MX Pro software (Stratagene) as recently described [14]. The reactions were carried out in 96-well polypropylene plates covered with optical caps (Stratagene). The plates contained triplicates of each cDNA sample and no-template controls with water instead of cDNA templates. PCR products were sequenced to evaluate specificity of the primer pairs. The 15-μL reaction mix contained 5 μL cDNA, 12.5 μL Brilliant SYBR Green QPCR Master Mix (Fermentas) with 300 nM of each primer. Cycling conditions were 10 min at 95°C, followed by 40 cycles of 30 s at 95°C, 1 min at 58°C, and 30 s at 72°C. The cDNA of all samples were amplified on the same plate for every primer pair to ensure equal amplification conditions. Specificity of amplification products was confirmed by melting curve analyses. For each sample, results were documented as cycle threshold (threshold set to 100 relative fluorescence units) values of background subtracted qPCR fluorescence kinetics by using the MX Pro Stratagene analysis software, applying the adaptive baseline, amplification based threshold, and moving average algorithm enhancement.

**Table 2:** Primer Sequences of used for qPCR

Gene	Primer sequence (5' - 3')	Amplicon size	**Accession no**.
HEPACAM1	fw-CACTGGAGAGAAGACCATCAA rev- CTCGTGGGAGCAGTTGAG	120 bp	ENSCAFT00017511
HEPACAM2	fw-GCTCCAGTGACCAAAGAAGA rev- CTCGAAGTCCATAAGGTCCA	117 bp	ENSCAFT0003183
HPRT	fw-TGCTCGAGATGTGATGAAGG rev-TCCCCTGTTGACTGGTCATT	191 bp	NM_000194
A5B	fw-GCACGGAAAATACAGCGTTT rev-TTGCCACAGCTTCTTCAATG	186 bp	NM_001686
RP32	fw-ATGCCCAACATTGGTTATGG rev-CTCTTTCCACGATGGCTTTG	180 bp	XM_540107

### Quantification of target gene expression

mRNA expression levels of the target genes (TG) were quantified using the ΔΔCT-method with multiple housekeepers as previously described [15,16]. Housekeeper genes were selected from a panel of reference genes (RG) according to the GeNorm algorithm and represent the best reference genes for canine mammary gland. Fold change (FC) in TG expression levels in the sample of interest (SOI) was normalized to geometric mean of housekeeper gene expression and relative to the normal mammary gland epithelium (NG). Finally the FC of GOI expression was calculated with the ΔCT_GOI _multiplied by the normalization factor. Specifically, gene expression levels of all adenomas, carcinomas and lymph node metastases (SOI) was normalized to RG expression and compared to NG of the same dog. Normal distribution and significance of differences in mRNA expression levels in the different tissues were confirmed by Kolgomorow-Smirnow-Test and univariate ANOVA.

### Immunohistochemistry

HEPACAM1 expression was immunohistochemically detected using the ABC-method. HEPACAM1 specific antibody (pab, 1:150; Sigma, HPA017613) was diluted in Tris-buffered saline (TBS, 50 mM, pH 7.6) and incubated at 4°C overnight after a blocking step with 50% goat serum in TBS (30 min at room temperature). Detection of HEPACAM1 required antigen retrieval by citrate pre-treatment for 30 min at 96°C. Goat anti-rabbit IgG (pab, 1 in 200; Vector, BA1000) was used as secondary antibody. Diaminobenzidine tetrahydrochloride (Sigma Aldrich) was used as chromogen and slides were counterstained with hematoxylin. As positive control paraffin embedded human liver specimen was used. As negative control, sections were incubated with an unspecific immune serum. No unspecific staining was detected in any tissue examined. Tissues were evaluated in 10 random 400× magnification fields in three serial sections and graded negative if less than 10% of the cells in a tumour were immunohistochemically positive.

## Authors' contributions

All authors read and approved the final manuscript. RK conceived the study, carried out the laser microdissection, quantitative RT-PCR, statistical analysis and drafted the manuscript. AC and PK carried out the immunohistochemical staining and analysis. LB helped in selection and collection of fresh frozen tissue samples of canine mammary tumors and clinical information. ADG conceived the study and helped to draft the manuscript.
